# Optimizing Outcomes in Immunocompromised Hosts: Understanding the Role of Immunotherapy in Invasive Fungal Diseases

**DOI:** 10.3389/fmicb.2015.01322

**Published:** 2015-11-26

**Authors:** Sharada Ravikumar, Mar Soe Win, Louis Yi Ann Chai

**Affiliations:** ^1^Division of Infectious Diseases, University Medicine Cluster, National University Health System, Singapore, Singapore; ^2^Yong Loo Lin School of Medicine, National University of Singapore, Singapore, Singapore

**Keywords:** invasive fungal infections, immunocompromised, immune regulation, immune enhancement, cytokines

## Abstract

A major global concern is the emergence and spread of systemic life-threatening fungal infections in critically ill patients. The increase in invasive fungal infections, caused most commonly by *Candida* and *Aspergillus* species, occurs in patients with impaired defenses due to a number of reasons such as underlying disease, the use of chemotherapeutic and immunosuppressive agents, broad-spectrum antibiotics, prosthetic devices and grafts, burns, neutropenia and HIV infection. The high morbidity and mortality associated with these infections is compounded by the limited therapeutic options and the emergence of drug resistant fungi. Hence, creative approaches to bridge the significant gap in antifungal drug development needs to be explored. Here, we review the potential anti-fungal targets for patient-centered therapies and immune-enhancing strategies for the prevention and treatment of invasive fungal diseases.

## Introduction

From among more than a million species of fungi present in nature, only a few 100 of them are capable of causing infections in humans (O’Brien et al., 2005). Of these, only a handful can cause diseases in healthy people, which is mostly superficial in nature (Kohler et al., 2015; LIFE at www.life-worldwide.org). Invasive fungal diseases (IFD) usually occur in susceptible individuals who are immunocompromised due to serious illnesses such as leukemia, neutropenia, AIDS, etc. In addition, medical advances have created vulnerable populations such as patients undergoing chemotherapy, solid and hematopoietic stem cell transplantation (HSCT), complex surgeries, immunosuppressive therapies for auto-immune and auto-inflammatory diseases, antibiotic therapies and treatment in intensive care units.

The major fungi responsible for these invasive infections, which kill about one and a half million people every year, are *Candida*, *Aspergillus*, and *Zygomycetes* species. Invasive candidiasis is the fourth and sixth most common nosocomial infection in US and Europe respectively with a high mortality rate ranging from 36 to 63% (Wisplinghoff et al., 2004; Brown et al., 2012; Rodrigues et al., 2014). The risk factors for candidemia include prior antibiotic usage, abdominal surgery, *Candida* colonization, central lines and parenteral nutrition (Wey et al., 1989; Blumberg et al., 2001) *Aspergillus* is a ubiquitous filamentous saprophytic mold whose conidia are dispersed in the air. Like the Zygomycetes, these molds cause several invasive diseases in hosts with markedly suppressed immunity and have a mortality rate in excess of 50–60% despite treatment (Herbrecht et al., 2002; Neofytos et al., 2009).

Such is the concern of the impact of IFD in immuno-compromised patients. This is despite the ever wider availability of anti-microbials beyond the conventional amphotericin-B based preparations and in the recent decade especially, the newer generation and classes of anti-fungals like voriconazole, posaconazole, and isavuconazole (of the azole family) and the echinocandins (caspofungin, anidulafungin, and micafungin). Some of the reasons for the high mortality are the difficulties in the early and correct diagnosis of invasive fungal infections (de Pauw and Picazo, 2008; Erjavec et al., 2009) as well as drug resistance profiles among specific fungal pathogens (Perlin, 2007; Verweij et al., 2007; Xie et al., 2014). The main reason for the poor outcomes from invasive disease nonetheless, is the incapacity of the patient’s compromised immune system to respond appropriately to the invading pathogen despite the presence of antimicrobials.

The response to such a challenge faced by the clinician at the bedside has led to exploration of novel therapeutic modalities beyond conventional antimicrobials; specifically, the manipulation and augmentation of the host immune response in the face of IFD. Through understanding how the immune system can detect the fungi, immunotherapeutic strategies may be formulated as adjuncts in the management of IFD.

## Immune Recognition and Response by the Host

The susceptibility and outcome of fungal infections depend on two main factors: the pathogen and the host. Pathogen factors may include the dose of the infecting fungi and its virulence. The efficacy of the immune response and the degree of the immune suppression in the patient are the major host determinants. The host defense capacity to fungal infection range from the protective mechanisms provided by skin, mucosa and innate immunity to the humoral response and adaptive immunity (Mueller-Loebnitz et al., 2013). The innate immune system despite its lack of specificity has been considered to bear significant importance in the defense mechanism against fungi. Monocytes, macrophages, neutrophils, and natural killer (NK) cells effect anti-fungal capabilities through phagocytosis, and directed pathogen killing. The fungal cell wall is the first structure encountered by host cells. Fungal cell wall is made up of various polysaccharides that have immune activating and modulatory properties. These pathogen associated molecular patterns (PAMPs); such as alpha and beta glucans, chitins, mannans, β- 1, 2-oligomannosides and galactomannan of varying constitutions in the cell wall of various fungi allow recognition by the innate immune cells; mainly monocytes, macrophages, dendritic cells (DCs) and endothelial cells (Netea et al., 2008). Pathogen recognition receptors (PRRs), a protein family of cellular receptors that mediate recognition of microbial pathogens and subsequent inflammatory response are present on the surface of DCs and macrophages (Hamad, 2012).

## Immune Recognition

One of the main PRRs are the Toll-like receptors (TLRs), whose role in the recognition of *Aspergillus* and *Candida* has been well documented especially, TLR2, TLR4, and TLR9 (Pasare and Medzhitov, 2005; Takeda and Akira, 2005; Goodridge and Underhill, 2008; Uematsu and Akira, 2008; Loures et al., 2010). The PRRs mounted on the host cells recognize specific fungi cell wall moieties of polysaccharide origin, namely the PAMPs. Fungal PAMPs for cell surface TLRs have been identified mainly through studies involving fungi with cell wall mutations. For instance, fungal phospholipomannans (PLMs), linear beta-1, 2-oligomannosides and glucuronoxylomannan (GXM) are known to bind with TLR2, while, O-linked mannans have been shown to activate TLR4 (Netea et al., 2006). Apart from cell surface PAMPs, nucleic acids released from the fungi in the phagosome also stimulate TLRs and modulate the host responses. TLR 9 activation occurs through interaction of genomic DNA whereas double stranded and single stranded RNA stimulate TLR3 and TLR7 respectively (Bourgeois and Kuchler, 2012).

Recognition of fungal antigen by TLR4 leads to pro-inflammatory cytokine production by NF-κB activation mediated by the adaptor protein Myd88. Bellocchio et al. (2004) supported that TLR4-mediated pro-inflammatory effects are protective against invasive aspergillosis by showing increased susceptibility of TLR4^–/–^ mice to *Aspergillus fumigatus* infection. Mutation of Asp299Gly in TLR4 is associated with increased incidence of pulmonary aspergillosis (Carvalho et al., 2008). It was subsequently demonstrated that HSCT patients in possession of the D299G/T399I haplotype were at higher risks of invasive aspergillosis (Bochud et al., 2008). TLR2 was shown to influence early recruitment and killing capacity of neutrophils against *A. fumigatus* (Bellocchio et al., 2004). TLR2^–/–^ mice infected intraperitoneally with *Candida albicans* were found to have lesser recruitment of neutrophils and monocytes (Tessarolli et al., 2010). However, TLR2^–/–^ mice had decreased fungal burden compared to the control mice accompanied by increased production of interleukin 12 (IL12) and decreased production of IL10. The role of TLR2 is still under debate as studies based on targeted patient genotype of TLR2 did not reveal enhanced susceptibility. TLR9^–/–^ mice are reported to have higher fungal burden than control mice and found to be producing more IL10 and lower IL12 which is in contrast to findings in TLR2^–/–^ mice. Mutations in TLR9 are associated with increased incidence of allergic bronchopulmonary aspergillosis (Carvalho et al., 2008; Mezger et al., 2010). An association of invasive aspergillosis was also seen in patients undergoing HSCT with SNPs in TLR1 and TLR6 (Kesh et al., 2005). It should be noted that TLR response may vary depending on fungal species and morphotype, and route of infection as well as the specific fungal infection (Romani, 2011).

Another family of PRRs that are important in the recognition of fungal PAMPs are C-type lectin receptors, otherwise known as CLRs. β-glucans present on the cell walls of *Candida* and *Aspergillus* species activate Dectin-1 receptor, while Dectin-2, and Dectin-3 mainly recognize hyphal α-mannan (Saijo et al., 2010). N-mannan is recognized by mannose receptor while galectin-3 binds to β-mannans. Fungal N- linked mannans also bind to DC-SIGN and mannose binding lectin (MBL) receptors present on phagocytes (Becker et al., 2015).

Dectin-1 is the most widely known CLR associated with fungal recognition. Dectin-1 recognition of β-glucan activates canonical and non-canonical NF-κB activation by two pathways, Syk-CARD9 and RAF pathways, resulting in increase in the pro-inflammatory cytokine production. Stimulation of Dectin-1 also increases IL1β and IL18 production through NLRP3 inflammasome pathway. Dectin-1 also collaborates with TLR2 to trigger pro-inflammatory cytokine production upon recognition of *Candida albicans* and zymosan. Dectin-1 deficient and CARD 9 deficient mice have predisposition to *Candida* infections (Ferwerda et al., 2009; Drewniak et al., 2013). Dectin-2 pairs with FcRγ to induce pro-inflammatory cytokine release. Dectin-1 plays an important role in human fungal infections too. It is evident from the polymorphism Y238X noticed in a Dutch family whose members were subject to recurrent vulvovaginal candidiasis and/or onychomycosis, while increased oral and gastrointestinal colonization of *Candida* was observed in HSCT recipients. In addition, it was noticed that there were defects in the expression of Dectin-1 and β-glucan recognition by phagocytes coupled with decrease in the production of cytokines, especially IL17 (Ferwerda et al., 2009; Plantinga et al., 2009). Similarly CARD9^–/–^ patients show increased susceptibility to chronic mucocutaneous candidiasis and reduced Th17 cells (Glocker et al., 2009). MINCLE, which is mainly expressed by macrophages, also induce NF-κB activation through Syk-CARD9 signaling. Mannose receptors are involved in the phagocytosis of un-opsonized *Candida* yeasts. Mannose receptor interacts with galectin-3, a PRR which recognizes carbohydrate moieties on fungal cell wall, to induce TNFα production (Esteban et al., 2011; Kawai and Akira, 2011).

Both *Candida* and *Aspergillus* also trigger an immune response through activation of the inflammasome—most well described through NLRP3 and caspase-1 activation, with the involvement of the tyrosine kinase Syk and Dectin-1 (Gross et al., 2009; Said-Sadier et al., 2010). The non-canonical caspase-8 pathway is also implicated in the context of *Candida* (Gringhuis et al., 2012). Both result in the cleaving and production of IL1β, a pivotal mediator of inflammatory response together with interferon gamma (IFNγ) and tumor necrosis factor alpha (TNFα). The invocation of a “pro-inflammatory” response necessitates a “counter-regulatory” component which is maintained by IL4 and IL10 and more recently, possibly through the inhibitory group of NLR (nucleotide-binding domain, leucine-rich repeat containing) proteins (Ting et al., 2010). It is believed that it is in the context of such a conventional paradigm of a balance between a “pro- and anti-inflammatory milieu” that host susceptibility and outcome of an IFD episode may be determined (Chai et al., 2011).

## Immune Regulation

### Role of Neutrophils

The state of neutropenia is a well-established risk factor for invasive aspergillosis (Marr et al., 2002). Neutrophils, being the primary effector cells of innate immune system, efficiently and rapidly kill fungi by various mechanisms. Neutrophils are capable of recognizing fungi by TLR2, TLR4, Dectin-1, and complement receptors such as CR1 and CR3 (Braem et al., 2015). MAP kinase signaling is reported to mediate neutrophil activation, especially ERK signaling pathway, since inhibition of ERK signaling pathway abolishes *C. albicans* induced neutrophil migration (Wozniok et al., 2008). Once activated, neutrophils are able to release neutrophil extracellular traps as well as an array of cytokines and chemokines. Neutrophils recruitment, activation and survival in inflammatory sites are affected by Th17 controlled pathway in fungal infections. Neutrophils are also the source of pattern recognition molecule, pentraxin 3 (PTX3) which forms complexes on the conidial surface of the fungus and acts as an opsonin, enhancing recognition and phagocytosis of conidia through mechanisms that depend on Fcγ receptor, CD11b and complement (Mantovani et al., 2011; Cunha et al., 2014).

### Role of Dendritic Cells/Monocytes/Macrophages

Dendritic cells serve the bridge between innate and adaptive immunity since they can present antigen to T cells, activate both innate and adaptive immune system by release of cytokines and chemokines. DCs can recognize fungal pathogens by the receptors such as Dectin-1, TLR2, and TLR4. Production of CCL20 as well as PTX3 increased with the activation of DCs (Mezger et al., 2008). DCs also mature after phagocytosis of fungal cells and promote the differentiation of naive T cells to CD4^+^ T cells which are essential for antifungal defense. *Aspergillus* conidia and hyphae induce NF-κB translocation and release of proinflammatory cytokine TNFα, and MIP2 in TLR2 and TLR4 dependent manner via adaptor protein Myd88.

Monocytes are macrophage and DC precursors; they serve as phagocytes as well as antigen presenting cells. Monocytes produce CCL20 which activates neutrophils, monocytes and naive T cells. Alveolar macrophages destroy *Aspergillus* conidia via non-oxidative mechanisms. The activity of macrophages can be enhanced by GM-CSF or IFNγ (Mueller-Loebnitz et al., 2013).

## Immune Resistance vs Immune Tolerance

T cells act as the immune modulators and master effectors in the immune response against fungal pathogens. Conventionally, Th1 response is associated with TLR4 signaling resulting in secretion of IFNγ and TNFα (for protection against fungal pathogen), while Th2 response is associated with TLR2 signaling resulting in the production of anti-inflammatory cytokines (IL4 and IL10) to regulate the inflammatory response (which unfortunately leads to more susceptibility against fungal infection). Th17 cells have been increasingly recognized to serve one of the central roles in the anti-*Candida* response especially the mucosal immunity. Th17 has long been attributed to autoimmune diseases while defective Th17 response results in mucocutaneous candidiasis in patients with primary immunodeficiencies (Zelante et al., 2009). In fungal infections, Th17 activation occurs through Syk-CARD9, Myd88 and mannose receptor signaling pathways in DCs and macrophages (Romani, 2011). Activation of IL17 results in the recruitment of neutrophils, defensins and ultimately results in inflammation. However, IL17 activation is also associated with high inflammatory pathology and inhibitory effects on the IFNγ related activation of indolamine 2, 3-dioxygenase (IDO) that is important for immune tolerance function (Romani and Puccetti, 2008). *Candida albicans* is known to dampen Th17 response resulting in chronic inflammation due to the impairment of IL17 dependent neutrophil recruitment leading to fungal persistence and immune dysregulation (Cheng et al., 2010).

While inflammation and immune response is necessary to eliminate the fungus, it is also important to limit the collateral damage to tissue and restore homeostasis to the environment. IL10, a major suppressive cytokine produced by CD4^+^ T regulatory cells plays an important role in keeping inflammation under control. However, the delicate balance of IL10/IFNγ needs to be in check since high level of IL10 suppresses the activity of IFNγ which provides the main Th1 defense against fungal infections. IDO which is a product of tryptophan metabolism is also increasingly recognized as the master regulator of immune resistance and tolerance since it can induce T regulatory cells and inhibit Th17. IDO and kynurenines balance immune tolerance and resistance by providing adequate elimination of fungal pathogen while preventing the unacceptable level of inflammation and allergy (Zelante et al., 2009).

## Immune Enhancement Strategies

**FIGURE 1:**
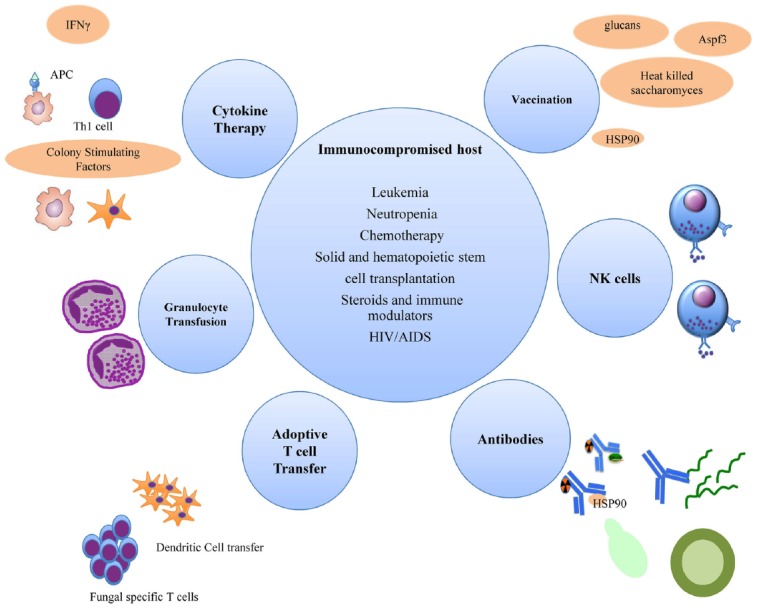
**Immune enhancement strategies for invasive fungal infections in immunocompromised hosts**.

The increased understanding of anti-fungal host responses has facilitated novel approaches into molecular and cell-based immunotherapeutics for invasive fungal infections (Figure 1). Notably, the major protective host response against fungi is the effective induction of Th1 and IFNγ responses, which in turn, activates effector phagocytic cells that kill the fungi. A cautionary note, however, is that this inflammatory response needs to be appropriately regulated or curbed when the pathogen or stimulatory ligand is contained, to minimize progression into a chronic inflammatory state which may induce collateral tissue damage.

### Cytokine Therapy

The use of recombinant cytokines such as human granulocyte macrophage colony-stimulating factor (GM-CSF), granulocyte colony-stimulating factor (G-CSF), macrophage colony-stimulating factor (M-CSF) and interferon-gamma (IFNγ) have been explored as immune enhancing agents. GM-CSF, G-CSF, and M-CSF belong to the family of hematopoietic cytokines. They stimulate the proliferation of granulocyte and/or macrophage progenitor cells, induce differentiation and maturation, and stimulate functional activity of mature hematopoietic cells (Pikman and Ben-Ami, 2012). GM-CSF alone or in combination with IFN-γ has been shown to enhance the fungicidal activity of innate phagocytic cells *in vitro* and *in vivo*. GM-CSF has been shown to preferentially enhance both the numbers and activity of type 1 DCs and cause upregulation of macrophage dectin-1 expression (Willment et al., 2003; Van de Veerdonk et al., 2010). Human M-CSF enhanced the activity of phagocytic cells and prolonged survival alone or in combination with amphotericin B in immunosuppressed mice with systemic *Candida* infection (Kuhara et al., 2000). Similarly, M-CSF when added to standard antifungal treatment of 46 stem cell transplantation recipients with progressive fungal infections showed better overall survival rates (Nemunaitis et al., 1993).

G-CSF is widely used in clinical practice during chemotherapy induced neutropenia. While G-CSF clearly reduces neutropenic days and neutropenia-related hospitalization, its efficacy in clinical outcomes including infection and mortality rates remain less clear (Smith et al., 2006). In a review of 925 mucormycosis cases, 15 of 18 patients showed favorable clinical response when given G-CSF adjunctive therapy (Roden et al., 2005). Clinical data on the use of GM-CSF as adjuvant antifungal therapy are scarce. Few case reports or small patient series with drug-refractory invasive aspergillosis infection have been published but provide limited information. Recently, a retrospective assessment of 66 patients was performed in whom GM-CSF was given during antifungal therapy to high-risk cancer patients and stem cell transplant recipients with IFD. A complete or partial response occurred in more than half of the patients treated with GM-CSF despite recent treatment with antineoplastic therapy and presence of other predictors of poor outcomes (Safdar et al., 2013). Further prospective studies to assess CSFs efficacy in the treatment of established fungal disease are needed.

IFNγ, produced by T and NK cells, increases the cytotoxic capacity of antigen presenting cells and intracellular killing. In a recent prospective case series, eight patients with invasive *Candida* and/or *Aspergillus* infections were treated with recombinant IFN-γ for 2 weeks in addition to standard antifungal therapy. Recombinant IFN-γ treatment in patients with invasive *Candida* and/or *Aspergillus* infections partially restored immune function, as characterized by an increased HLA-DR expression in those patients and an enhanced production of pro-inflammatory cytokines involved in antifungal defense (Delsing et al., 2014). IFNγ is also used in the treatment of recalcitrant aspergillosis (Kelleher et al., 2006; Bandera et al., 2008; Estrada et al., 2012). Further large-scale clinical studies to assess the potential clinical benefit of IFNγ is needed, but the cost of the drug remains a major concern.

Preclinical trials have assessed other pro-inflammatory cytokines that upregulate the antifungal Th1 response such as IL12, IL15, and TNFα as candidate adjuvants. IL12 is required for *Candida*-induced differentiation of Th1 cells *in vivo* (Romani et al., 1997) and for the antifungal activity of monocytes against *A. fumigatus* hyphae *in vitro* (Roilides et al., 1999). The usefulness of IL12 as immune enhancer is controversial. Invasive mold infections were reported in two autologous stem cell transplantation recipients treated with IL12 (Toren et al., 1997), raising concern that IL12 may paradoxically provoke an immune flare to fungal pathogens.

IL15 is also a potential new drug candidate. This cytokine, shares biological activities with IL2, in enhancing antifungal granulocyte activity in cell cultures (Vazquez et al., 1998; Winn et al., 2003). Neutralization of TNFα, a signature cytokine of Th1 cells, increases the susceptibility of mice to invasive aspergillosis, whereas intratracheal instillation of TNFα agonist peptides confers protection against *A. fumigatus* conidia (Mehrad et al., 1999). Further preclinical investigation is required not only for these cytokines, but also for IL18 and IL36 belonging to the interleukin 1 family (Gresnigt et al., 2013; Ketelut-Carneiro et al., 2015).

### Granulocyte Transfusion

Transfusion of granulocytes from healthy donors has been used anecdotally for immune enhancement in patients with neutropenia who suffer from invasive fungal infections. Earlier attempts were beset by the lower yield and quality of granulocytes recovered from steroid treated donors. However, with advances in apheresis methods, better sedimenting agents and the recent use of recombinant cytokines like G-CSF and IFN-γ1b in addition to steroids, the yield and quality of leukocytes from healthy donors have improved.

The efficacy of granulocyte transfusion has been shown by the increased survival rates following its use in the treatment of cancer patients with candidemia (Price et al., 2000). In an uncontrolled prospective study of 23 patients treated for IFD with granulocyte transfusion, no recurrent infection was observed (Mousset et al., 2005). However, in a Phase III randomized trial of 74 patients with febrile neutropenia, 55 of whom had IFD and 39 had received stem cell transplantation, there was no clear effect of granulocyte transfusion on survival up to day 100 (Seidel et al., 2008). Though major randomized trials are lacking for patients with invasive aspergillosis and mucormycosis, good clinical efficacy and safety using appropriate granulocytes is evident through various small case series and case reports (Dignani et al., 1997; Illerhaus et al., 2002; Slavin et al., 2002; Safdar et al., 2006). Therefore, the use of granulocyte transfusions in patients with severe neutropenia and uncontrolled infection, in spite of appropriate antifungal therapy might be considered as a potential life-saving treatment option.

### Antibodies

The era of antibody-based therapy for invasive fungal infections dawned with the discovery of protective monoclonal antibodies (mAbs) against the capsular polysaccharide of *Cryptococcus neoformans* (Dromer et al., 1987). Subsequently, protective antibodies against *Candida albicans* (Han and Cutler, 1995; Moragues et al., 2003), *Aspergillus fumigatus* (Chaturvedi et al., 2005) and other fungi were elucidated.

Two antifungal mAbs have been evaluated in clinical trials. 18B7, a mAb against the capsular polysaccharide of C. neoformans was found to be safe in a Phase I study (Larsen et al., 2005) but there is a lack of efficacy data. Efungumab (Mycograb) is a genetically engineered human recombinant antibody against fungal heat shock protein 90. HSP90 is an immunodominant antigen of the *Candida* cell wall and is required for its survival. In preclinical studies, Mycograb showed activity against a wide range of *Candida* species and synergized with antifungal drugs (Matthews et al., 2003; Hodgetts et al., 2008). But the role of Mycograb at the bedside remains still controversial. Results of a double-blind clinical trial in 117 patients with invasive candidiasis receiving liposomal Amphotericin B with or without Mycograb, showed that by day 10, the patient group receiving Mycograb combination (84 vs 48%; *p* < 0.001) had complete response with more rapid clearance of fungal cultures and reduced *Candida*-attributable mortality rate (Pachl et al., 2006). However, due to methodological and safety issues (Herbrecht et al., 2006), the drug has not gained licensure yet.

On a similar note, monoclonal antibodies mAb C7 and mAb A9, against *Candida* cell wall mannoprotein and *A. fumigatus* cell wall glycoprotein respectively, exhibit direct fungicidal activity (Moragues et al., 2003; Chaturvedi et al., 2005) with reduced fungal burden and increased survival rate in murine models of invasive infection.

Killer anti-idiotypic antibodies, which mimic broad spectrum antimicrobial peptides have been developed. These antibodies, upon intranasal administration to immunosuppressed mice with invasive aspergillosis have resulted in cure and long-term survival (Cenci et al., 2002).

Radioimmunotherapy is another novel antibody-based concept, whereby radiolabeled antibodies that recognize fungal antigens are used to deliver microbicidal radiation with less systemic toxicity (Bryan et al., 2010). It is hoped that radiolabeled mAbs that bind antigens shared by many pathogenic fungi, such as HSP60 and β1, 3 glucan, may act as adjuncts in tandem with conventional antifungals (Bryan et al., 2012).

### Vaccination

Antifungal vaccines is an area that has drawn increasing interest and research in recent years. The effective usage of fungal vaccines is limited in the immunocompromised hosts as they not only tend to mount weak protective responses to vaccines but are also at risk from live attenuated formulations. Hence fungal vaccines are often based on standardized cellular subunits which require an adjuvant to induce protective immunity. Heat shock proteins may serve as powerful adjuvants while the immune response may be enhanced by mannosylation of antigens (Spellberg, 2011). Protective immunity arises from both T-cell responses, specifically Th1 and/or Th17 (Wuthrich et al., 2011) and antibody responses.

Preclinical evaluation of vaccines to a number of important fungal pathogens have been performed and at least two have been subject to Phase I clinical trials (Pikman and Ben-Ami, 2012). Universal fungal vaccines may be on the horizon with a conjugate vaccine that evokes antibodies to β-glucans offering cross-protection against three major fungal pathogens: *C. albicans*, *A. fumigatus*, and *C. neoformans* (Torosantucci et al., 2009). Another promising panfungal vaccine preparation originates from heat-killed *Saccharomyces* and is found to confer protection against *Aspergillus*, *Coccidioides*, and *Candida* infections (Stevens et al., 2011).

Though animal studies with crude *A. fumigatus* antigens are promising, the ideal dose that can be safely administered to humans is not well understood (Stevens, 2004). Vaccination of mice with a distinct *Aspergillus* antigen Aspf 3 prior to immunosuppression was shown to confer protection against subsequent inhalational challenge with *A. fumigatus* (Ito et al., 2006). It was shown that immunization confers cellular rather than humoral immunity since naive mice were protected from invasive aspergillosis by passive transfer of CD4^+^ cells rather than anti-Aspf 3 antibodies from immunized mice (Diaz-Arevalo et al., 2011). Additional vaccine candidates include secreted protein Pep1p and anchored proteins Gel1p and Crf1p (Bozza et al., 2009) of which, Crf1p proved to be immunogenic with cross-reactivity and protection against *C. albicans* (Stuehler et al., 2011).

### Natural Killer Cell Treatment

Recently the role of NK cells in antifungal immunity is being investigated. It has been found that IL2-primed NK cells are cytotoxic toward *A. fumigatus* germlings and hyphae, an effect that is not mediated through degranulation of its cytotoxic proteins like perforin, granzymes etc., but mediated by IFNγ and TNFα secretion (Bouzani et al., 2011; Schmidt et al., 2011). NK cells have been shown to be the most important source of IFN-γ in the lungs of neutropenic hosts during the early stages of invasive aspergillosis (Park et al., 2009). It was also shown that the chemokine ligand MCP1/CCL2 mediates recruitment of NK cells resulting in more rapid clearance of *Aspergillus* from the lungs (Morrison et al., 2003) implicating the potential for NK-based therapeutic applications.

### Adoptive T cell Transfer

Defective T-cell immunity is a hurdle in the path to a robust immune response to vaccines and antimicrobial treatment. Conceptually, this problem could be overcome by T-cell-independent vaccination, wherein the CD4^+^ T-cell-derived factor CD40L, required for DC costimulation of B cells, is replaced (Zheng et al., 2005).

One of the strategies to reduce the risk of invasive aspergillosis is the induction of Th1-type immune response that may be achieved by either transferring *Aspergillus*-specific Th clones or DCs that have been primed to trigger *Aspergillus*-specific immunity (Pikman and Ben-Ami, 2012). Adoptive T-cell transfer has been shown to decrease galactomannan levels significantly with higher survival rates as compared with patients who did not receive immunotherapy (Perruccio et al., 2004). Specific *Candida* cell wall proteins expressed during invasive infection have been synthesized as immunogenic peptide epitope–β-mannan conjugates. DCs pulsed with three of these epitopes conferred protection against disseminated candidiasis in mice. Of note is one epitope, derived from fructose-bisphosphate aldolase, which was shown to induce robust antibody dependent protective responses to *C. albicans* (Xin et al., 2008).

Various vaccine formulations using DCs to induce adoptive immunity to *Aspergillus* have been studied. DCs pulsed with live conidia, transfected with conidial RNA or primed with unmethylated CpG oligodeoxynucleotides and pulsed with Aspf16 antigens trigger specific Th1-type responses and protective immunity against invasive aspergillosis in a mouse model. DC infusion was shown to be more effective and superior to that of *Aspergillus*-specific T cells (Bozza et al., 2002, 2003). Subsequently, it was shown that DCs transfected with IL-12 DNA and pulsed with heat-inactivated *A. fumigatus* induced protective immunity against invasive pulmonary aspergillosis, as reflected by decreased fungal burden and increased survival (Shao et al., 2005).

## Conclusions and Future Perspectives

Despite the advances in our knowledge and understanding in pathogenesis, IFD continues to result in significant morbidity and mortality in immunocompromised patients. The current conventional therapeutic modalities have not been fully effective. In addition, prolonged use of antifungal agents pose the risk of emergence of fungi resistant to conventional drugs.

The urgent need of the hour is to improve treatment options for patients with IFD by the usage of newer and more effective drugs, alone or combined together that can cure the infection. The other promising solution would be the use of immunotherapeutic modalities to improve and enhance the host defense system against fungal pathogens. The increase in knowledge of the pathogenesis of fungal infections has ushered in a new era of immunotherapeutic options. It is of utmost importance that further relevant clinical trials be conducted to explore the various immunotherapeutic strategies that hold promise for the better treatment and control of IFD in the near future.

### Conflict of Interest Statement

The authors declare that the research was conducted in the absence of any commercial or financial relationships that could be construed as a potential conflict of interest.
